# Adsorption behavior of Mo(VI) from aqueous solutions using tungstate-modified magnetic nanoparticle

**DOI:** 10.1007/s11356-024-32251-y

**Published:** 2024-02-14

**Authors:** Elsayed M. Abu Elgoud, Ahmed I. Abd-Elhamid, Hisham F. Aly

**Affiliations:** 1 Nuclear Fuel Chemistry Department, Hot Laboratories Center, Egyptian Atomic Energy Authority, Cairo, 13759 Egypt; 2 Composites and Nanostructured Materials Research Department, Advanced Technology and New Materials Research Institute (ATNMRI), City of Scientific Research and Technological Applications (SRTA-City), New Borg Al-Arab 21934, Alexandria, Egypt

**Keywords:** Molybdenum, Tungstate, Magnetic nanoparticles, Adsorption, Selectivity

## Abstract

A new magnetic nanoparticle modified with sodium tungstate (Mnp-Si-W) was synthesized and employed for the sorption of molybdenum from aqueous solutions. The prepared nanoparticles (Mnp-Si-W) were characterized by different advanced techniques. Different parameters that influenced the adsorption percent of Mo(VI) were investigated using a batch process. Based on a systematic investigation of the adsorption isotherms and kinetics models, Mo(VI) adsorption follows the Langmuir model and pseudo-second-order kinetics. According to the Langmuir isotherm model, the Mnp-Si-W nanoparticles exhibited a maximum adsorption capacity of 182.03 mg g^−1^ for Mo(VI) at pH 2.0. The effect of competing ions showed that the prepared nanoparticles have a high selectivity for the sorption of molybdenum. Moreover, the effect of some interfering anions on Mo(VI) ion sorption is found in the following order: phosphate < sulfate < chromate. Finally, the nanoparticle (Mnp-Si-W) can be successfully reused five times.

## Introduction

Radioisotopes play a significant role in the peaceful uses of atomic energy. The radioisotope most widely applied in medicine is technetium-99 m, used in more than half of all nuclear medicine procedures. Technetium-99 m can be separated from its main parent ^99^Mo by physical or chemical separation processes (Tkac et al. 2018). This system makes feasible the use of short-lived ^99m^Tc even in places far away from ^99^Mo producing centers. Generally, two methods have been used for ^99^Mo production. Firstly is the neutron activation of molybdenum with natural isotopic composition or enriched in ^98^Mo (El Abd 2010; Uddin et al. 2015). Secondly is the neutron-induced fission of U-235 followed by the chemical separation of molybdenum-99 from uranium and other fission products (Rizk et al. 2018). Molybdenum is one of the strategic metals used in several applications including the steel industry as an alloying agent, thermo-couples, heat-resistant materials, the petrochemical sector as a catalyst, semiconductor, anticathodes of X-ray tubes, vacuum tubes, radios, optoelectronic industries, electron tubes, and fuel cladding (Tsai et al. 2016; Zeng and Cheng 2009; Xiong et al. 2011a, b; Gustafsson 2003). Furthermore, it has been proposed that advanced U–Mo nuclear fuel might be created by alloying uranium with molybdenum (Emam et al. 2023). Molybdenum has eight oxidation states (− 2, 0, 1, 2, 3, 4, 5, 6), but the oxidation states (− 2, 0, 1) are rare. In toxic natural water, + 6 is the predominant oxidation state (Du et al. 2003; Wang et al. 2009). There are seven stable isotopes of molybdenum: ^100^Mo, ^98^Mo, ^97^Mo, ^96^Mo, ^95^Mo, ^94^Mo, and ^92^Mo, with natural abundances of 9.6%, 24.1%, 9.5%, 16.6%, 15.9%, 9.2%, and 14.7%, respectively. Radioisotopes of molybdenum(VI) are one of the major products of spent nuclear fuels and the high-level radioactive liquid waste generated from nuclear power productions (Hassanpour and Taghizadeh 2016). The recovery and separation of molybdenum(VI) are desired for the processing of the nuclear fuel cycle. Therefore, it is a very important issue to develop a new adsorbent that can separate or adsorb molybdenum. Various adsorbents have been applied for the recovery and sorption of Mo(VI) and other heavy metals (Baral et al. 2006, 2007, 2009). For example, molybdenum removal from aquatic solutions using aluminum oxide was studied by Kurmysheva et al. (2023). According to their investigation, aluminum oxide has a high sorption capacity of 31.0 mg g^−1^ for molybdenum. Wu et al. (2021) employed chitosan-coated zirconium–iron sulfide composite for the effective sorption of Mo(VI) from aqueous solutions. They reported that Mo(VI) possesses a high adsorption capacity of 120.80 mg g^−1^. Gamal et al. (2021) used a synthetic magnetic chromium ferrite nanocomposite to study the sorption of Mo(VI). According to their investigation, magnetic Cr-ferrite has a maximal Mo(VI) adsorption capability of 26.8 mg g^−1^ at pH = 3.6. Chao et al. (2020) prepared chitosan-modified magnetic nanoparticles (Fe_3_O_4_/CTS) for the removal of Mo(VI) from aqueous solutions. They reported that the best conditions for maximum removal of Mo(VI) are an adsorbent dosage of 0.40 g, 10 min of shaking time, and Mo(VI) solution in 100 mL with a pH of 4.0. Rizk et al. (2018) investigated the separation of Mo(VI) from certain fission products using perlite impregnated with trioctylamine. According to the experimental data, perlite impregnation increased the sorption efficiency of Mo(VI) from 3.6 to 93.2%. Marković et al. (2017) demonstrated the selective adsorption of molybdenum and rhenium by using magnetic glycidyl methacrylate. They found that at pH 2, 98% of molybdenum and 92% of rhenium were removed. Hassanpour and Taghizadeh (2016) utilized methacrylic acid (MAA), Mo(VI), ethylene glycol dimethacrylate (EGDMA), and Fe_3_O_4_@SiO_2_ for the production of magnetic Mo(VI) ion-imprinted polymer for Mo(VI) separation. Their results reported that the ion-imprinted polymer possesses a maximum Mo(VI) adsorption capacity of 28.0 mg g^−1^. El-Din et al. (2021) used alginate/Lix-84 beads to adsorb and separate Mo(VI) from some rare earth elements. Their results showed that Mo(VI) has a maximum sorption capacity of 72.2 mg g^−1^. The performance of TVEX-TOPO resin for the adsorption of Mo(VI) from nitrate medium was evaluated by Masry and Daoud (2021). Their studies indicated that the maximum sorption capacity toward Mo(VI) is 17.60 mg g^−1^. Metwally and Attallah (2019) used chabazite modified with N-methylene aniline (Ch-NMA composite) to study the adsorption of Mo(VI) radioisotopes from an aqueous solution. Their work reported that the Ch-NMA composite possessed a maximum sorption capacity of 29.7 mg g^−1^ for Mo(VI) at pH 3.0. Tungstate (WO_4_^2-^) is an anionic species that contains tungsten in its highest oxidation state (+ 6). It possesses unique chemical properties, including strong coordination abilities and redox activity. These properties make tungstate suitable for certain applications. Recently, several tungstate-based nanoparticles have been reported (Moghaddam et al. 2018; Dutta et al. 2014, 2015; Singh et al. 2013). Several composite adsorbents have been revealed in previous studies based on tungstate. For example, Wang et al. (2023) employed magnetic adsorbent based on ammonium phosphomolybdenum heteropoly tungstate for selective and effective sorption of Rb(I) and Cs(I). Their results demonstrated that Rb(I) and Cs(I) possessed maximum adsorption capacities of 0.78 and 0.85 mmol g^−1^, respectively. The removal of Pb(II) from aqueous solution by using graphene oxide–bismuth tungstate (GO–Bi_2_WO_6_) nanocomposite has been evaluated by Saadati et al. (2023). They declared that Pb(II) has a high adsorption capacity of 128.0 mg g^−1^. Graphene oxide–tungsten oxide (GO–WO_3_) was synthesized by Mashhoor et al. (2023) for the adsorption of Cu(II). According to their studies, Cu(II) possesses an adsorption capacity of 143.0 mg g^−1^. Tungsten and molybdenum exhibit similar chemical characteristics and belong to the same group in the periodic table. This similarity can be utilized to developed a material that can effectively interact with molybdenum ions. Therefore, the main objective of this work is to synthesize a new tungstate-modified magnetic nanoparticle for the selective adsorption and separation of molybdenum from aqueous solutions. The characterization of the prepared composite was done by using Fourier transform infrared spectroscopy (FTIR), transmission electron microscope (TEM), Brunauer–Emmett–Teller (BET), energy-dispersive X-ray (EDX) mapping, and X-ray diffraction (XRD). The kinetic, isotherm, and thermodynamic properties of the adsorption process were investigated to further comprehend the adsorption mechanism. Finally, the separation and recovery of Mo(VI) from certain fission product components were assessed.

## Experimental

### Materials and chemicals

The materials and chemicals used in this investigation were all of the analytical grades and applied without purification. These included FeCl_2_·4H_2_O (99%, Sigma-Aldrich), FeCl_3_·6H_2_O (99%, Loba Chemie), NH_4_OH (99%, Across), cetyl trimethyl ammonium bromide (CTAB) (99%, from Winlab, UK), tetraethyl orthosilicate, TEOS (99%, Sigma-Aldrich), methanol (99.9%, International Co. for Supp. & Med. Industries), sodium tungstate dehydrate (Na_2_WO_4_·2H_2_O, > 98% from Sigma-Aldrich), HNO_3_ (Sigma-Aldrich), and sodium molybdate obtained from Loba Chemical Co., India.

### Preparation of magnetic nanoparticles

Magnetic nanoparticles were prepared as previously reported (Abd-Elhamid et al. 2024a). Briefly, definite amounts of FeCl_2_·4H_2_O (1.6 mmol) and FeCl_3_·6H_2_O (2.8 mmol) were dissolved in a specific volume of double-distilled water (400.0 mL) and stirred at 80 °C to yield a clear solution. A definite volume of NH_4_OH (40 mL) was rapidly added to the previously iron solution to produce a brown suspension of magnetic nanoparticles. Afterward, the magnet was removed, and the suspension was left to cool. The magnetic nanoparticles were separated using a centrifuge, washed with double-distilled water multiple times, air-dried, and finally stored for future use.

### Preparation of silica-coated Mnp

One and a half grams of Mnp was dispersed in 4.0 L of double-distilled water at 105 °C using magnetic stirrer. In a separate beaker, the mixture of 50.0 mL TEOS and 1.0L MeOH was added to the magnetic nanoparticle suspension. Thereafter, 5.0 g of CTAB was put to the previous mixture and left stirred for another 1.0 h till complete dissolution. After that, 20.0 mL of NH_4_OH was added to the suspension and left stirred for 24 h at 105 °C. The resulting precipitate was collected by an external magnate, washed three times with water, and dried at 70 °C. Finally, the brown powder was air-burnt at 500 °C for 5.0 h to remove the surfactant.

### Modification with tungstate

Two grams of the burnt magnetic powder was dispersed in 1.0 L of double-distilled water to form a homogenous suspension. Amino silane, 10 mL, was applied dropwise to the solution and heated to 120 °C for 3.0 h to generate surface amino functional groups. The modified nanoparticles were collected with an external magnate and washed with water. The resulting powder was added to 1.0 L of double-distilled water containing 10.0 g Na_2_WO_4_ and stirred for 24 h at 180 °C. The resultant modified nanoparticles were collected by an external magnet, washed multiple times with distilled water, dried at 70 °C, and then kept for further use.

### Characterization and analysis

TEM, EDX, BET, XRD, and FTIR were used to characterize magnetic nanoparticles modified with tungstate both before and after sorption. Using a JEM-1010 device, a transition electron microscope analysis was carried out (JEOL Ltd., Tokyo, Japan). The USA-made PerkinElmer 1600 FTIR spectrometer was used to set the FTIR spectra. Using an Oxford energy-dispersive X-ray spectrometer, the elemental composition of Mo(VI) sorption on tungstate-modified magnetic nanoparticles was revealed (Oxford Link ISIS, Japan). XRD patterns were made by an X-ray diffractometer (XD-Dl, Kyoto, Japan). The Brunauer–Emmett–Teller surface area and pore size distributions were measured by measuring N_2_ gas adsorption–desorption (Nova 1000e Series, USA). All spectrophotometric measurements were conducted using a Shimadzu UV–visible double beam spectrophotometer, model UV-160A, from Japan. Inductively coupled plasma optical emission spectroscopy (ICP-OES, Shimadzu Sequential Type, Kyoto, Japan) was used to measure the interfering ions.

### Batch adsorption studies

A stock solution of molybdenum(VI) (1.0 g L^−1^) was prepared in 100.0 mL by dissolving 0.252 g of sodium molybdate in deionized water. The Alizarin Red S technique was used to spectrophotometrically determine the Mo(VI) concentration in the individual samples (Marczenko 1976). In a thermostated shaker bath (G.F.L. 1083, Germany) set to 25 °C, batch sorption studies were conducted by shaking 5.0 mL of a solution containing 100.0 mg L^−1^ Mo(VI) with 5.0 mg of the tungstate-modified magnetic nanoparticle. The batch system was conducted as presented in Table 1. The adsorption experiments were performed in triplicate. Subsequently, average values were computed based on the results obtained from these multiple trials.

**Table 1:** Experimental condition for sorption of Mo(VI) onto tungstate-modified magnetic nanoparticles (Mnp-Si-W)

Batch sorption study					
Effect of different parameters	pH	Shaking time, min	[Mo(VI)], mg L^−1^	Adsorbent dosage, mg	Temperature, K
Effect of solution pH	**1, 2, 3, 4, 6, 8, and 10**	60.0	100.0	5.0	298
Effect of shaking time	2.0	**5, 10, 15, 30, 60, and 90**	100.0	5.0	298
Effect of molybdenum concentration	2.0	30.0	**100, 200, 400, 600, 800, and 1000**	5.0	298
Effect of adsorbent dosage	2.0	30.0	100.0	**5, 10, 15, 20, and 25**	298
Effect of solution Temperature	2.0	30.0	100.0	5.0	**298, 308, 318, 328, and 338**

The following equation was used to calculate the adsorption capacity, *q*_e_, of tungstate-modified magnetic nanoparticle (mg g^−1^) (Elbarbary et al. 2023; Khalil et al. 2022).

1 $${q}_{{\text{e}}}={(C}_{{\text{o}}}-{C}_{{\text{e}}})\left(\frac{V}{m}\right) [\mathrm{mg }{{\text{g}}}^{-1}]$$

From the following relationship, the distribution coefficient (*K*_d_) of metal ions between the aqueous phase and the magnetic nanoparticle phase is calculated (Abu Elgoud et al. 2022a):

2 $${K}_{d}=\left(\frac{{C}_{o}-{C}_{e}}{{C}_{e}}\right)\left(\frac{V}{m}\right)$$

3 $${K}_{d}=\left(\frac{{q}_{e}}{{C}_{e}}\right)$$

Where *V* is the volume (L) and *m* is the weight (g) of the tungstate-modified magnetic nanoparticle; *C*_o_ and *C*_e_ are the initial and equilibrium concentrations (mg L^−1^) of Mo(VI), respectively.

The separation factor (*SF*) is determined as follows:

4 $${SF=K}_{{\text{d}}1}/{K}_{d2}$$

Where *K*_d1_ and *K*_d2_ indicate the Mo(VI) and other metal ion distribution ratios, respectively.

### Desorption investigations

The desorption behavior of tungstate-modified magnetic nanoparticles (Mnp-Si-W) was investigated to assess their reusability. The reagents used include sodium acetate, sodium hydroxide, and nitric acid. The desorption experiments were conducted by mixing 5.0 mg of the loaded Mnp-Si-W nanoparticles with 5.0 mL of the desorbing agent. The mixture was then shaken for 60.0 min at a temperature of 25 °C. After the shaking period, the Mnp-Si-W nanoparticles were separated from the solution by centrifugation and the concentrations of Mo(VI) in the desorbing solution (*C*_i_) were measured. The desorption efficiency (*DE*) is calculated using the following equation:

5 $$DE=\frac{{C}_{{\text{i}}}}{{C}_{{\text{o}}}} \times 100$$

In this equation, *DE* represents the desorption efficiency expressed as a percentage. *C*_i_ is the concentration of Mo(VI) in the desorbing solution after desorption, and *C*_o_ is the concentration of Mo(VI) within the Mnp-Si-W nanoparticles before desorption.

### Selectivity

In order to assess the selectivity of Mnp-Si-W nanoparticles towards Mo(VI), an experiment was conducted using a solution containing Y(III), Sr(II), Cs(I), Eu(III), and La(III) ions at a concentration of 100 mg L^−1^ from each. The solution also contained 0.05 g of Mnp-Si-W nanoparticles at a pH of 2. After an equilibrium time of 30.0 min, the Mnp-Si-W nanoparticles were separated from the solution using centrifugation. The remaining solution was then analyzed using ICP-OES (Shimadzu Sequential Type, Kyoto, Japan) to determine the initial and residual concentrations of Y(III), Sr(II), Cs(I), Eu(III), and La(III).

### Mathematical models

The non-linear kinetics and isotherm models used are tabulated in Table 2. The pseudo-first and pseudo-second-order equations were illustrated to study the sorption kinetics. The isotherm models applied to assure the experimental result include Langmuir, Freundlich, Temkin, and Dubinin–Radushkevich isotherm models.

**Table 2:** Non-linear forms of adsorption models

Isotherm	Non-linear form
Langmuir	${q}_{{\text{e}}}=\frac{{q}_{{\text{m}}}{K}_{{\text{L}}}{C}_{{\text{e}}}}{1+{K}_{{\text{L}}}{C}_{{\text{e}}}}$
Freundlich	${q}_{{\text{e}}}= {K}_{{\text{F}}} {C}_{{\text{e}}}^{1/n}$
Temkin	${q}_{{\text{e}}}=\frac{RT}{{b}_{T}{\text{ln}}{{A}_{{\text{T}}}C}_{{\text{e}}}}$
Dubinin–Radushkevich	${q}_{{\text{mDR}}}={e}^{-{\beta }_{{\text{DR}}}{\varepsilon }^{2}}$
Kinetic model	
Pseudo-first-order	${q}_{\mathrm{t }= }{{q}_{{\text{e}}}}_{({\text{cal}}.)}\left(1-{e}^{-{k}_{1}t}\right)$
Pseudo-second-order	${q}_{{\text{t}}}=\frac{{k}_{2}{q}_{{\text{e}}\left({\text{cal}}.\right)}^{2}t}{1+{k}_{2}{q}_{{\text{e}}}t}$

## Results and discussion

### Characterization

#### TEM

**Fig. 1:**
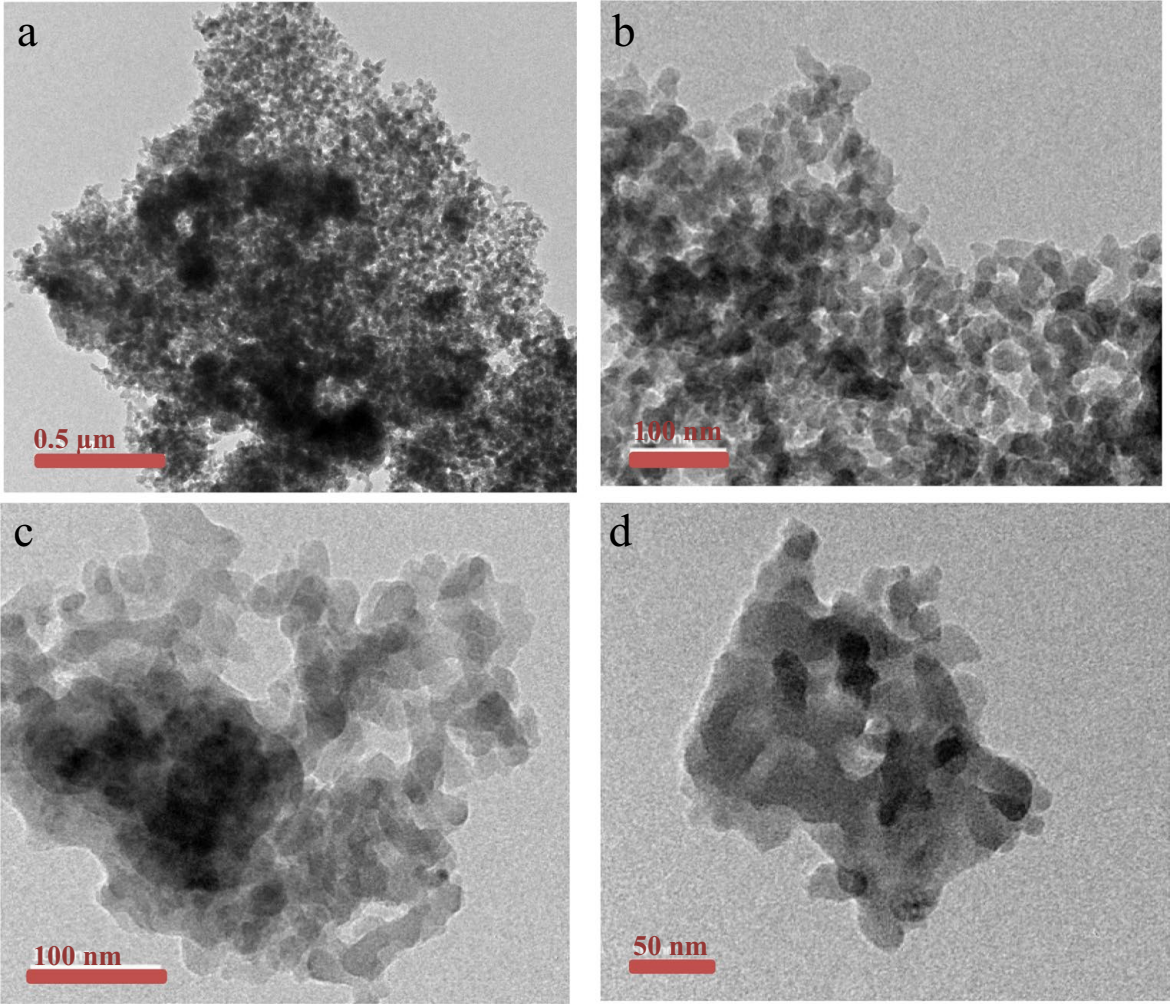
TEM images for tungstate-modified magnetic nanoparticles (Mnp-Si-W)

The TEM images of Mnp-Si-W are presented in Fig. 1. We noted that the tungstate-modified magnetic nanoparticles (Mnp-Si-W) were irregular spherical nanoparticles, and the nanoparticles have been agglomerated, which may have occurred during the drying stage.

#### EDX elemental mapping and analysis

**Fig. 2:**
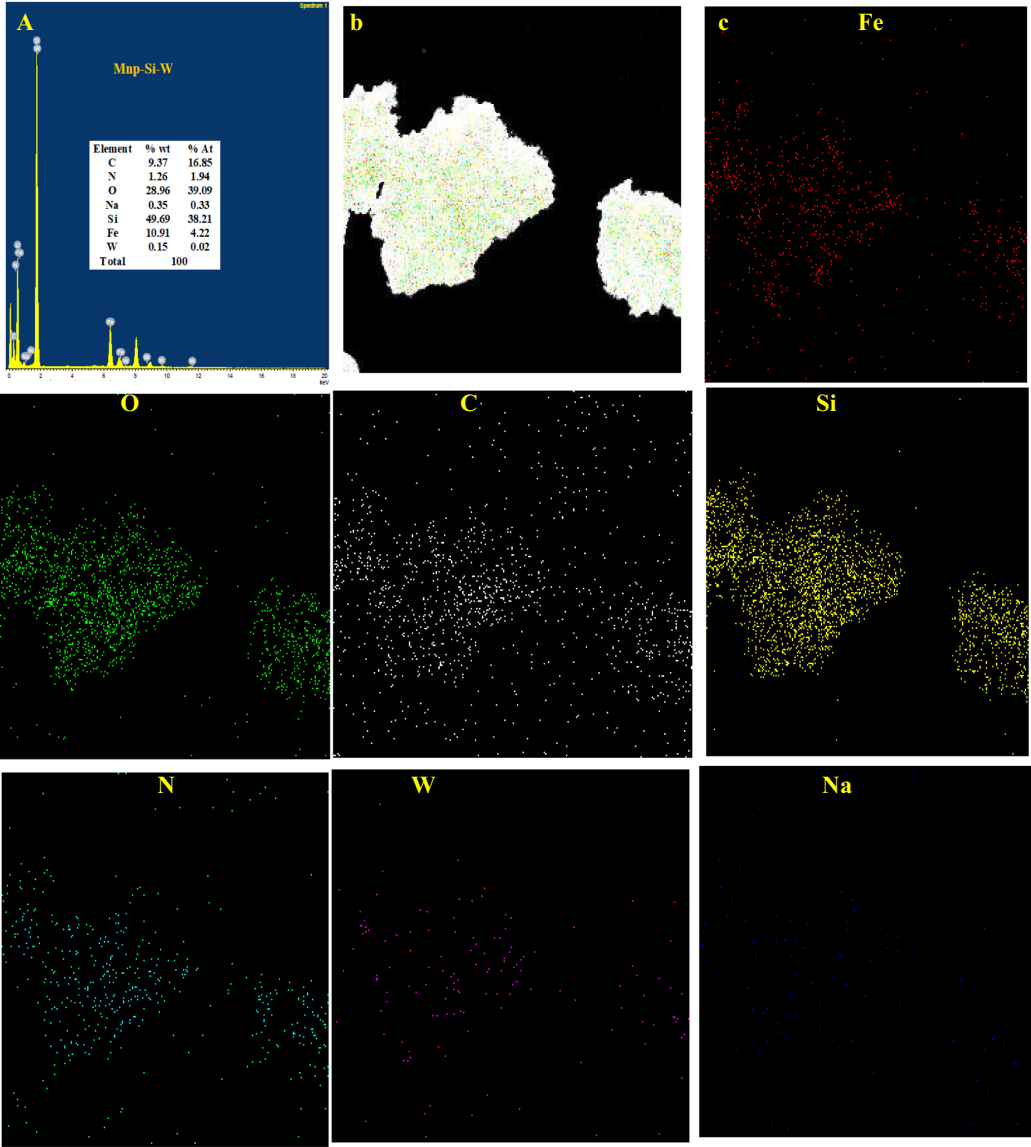
**a** TEM-EDS, **b** STEM image, and **c** EDX mapping of Mnp-Si-W nanoparticles

The TEM-EDS and TEM-mapping analysis could be considered as available devices to determine the elemental analysis and the distribution of the elements in the analyzed material. The TEM-EDS of the Mnp-Si-W was composed of C, N (amino silane), O (Mnp, SiO_2,_ and Na_2_WO_4_), Na (Na_2_WO_4_), Si (SiO_2_, amino silane), Fe (Mnp), and W (Na_2_WO_4_), which proved the preparation procedure (Fig. 2a). To further determine the distribution of various elements through the as-prepared nanoparticles, TEM mapping was investigated. The mapping analysis of Mnp-Si-W showed that the different elements that composed the nanoparticle were uniformly distributed in the nanoparticle, as shown in Fig. 2.

#### FTIR

**Fig. 3:**
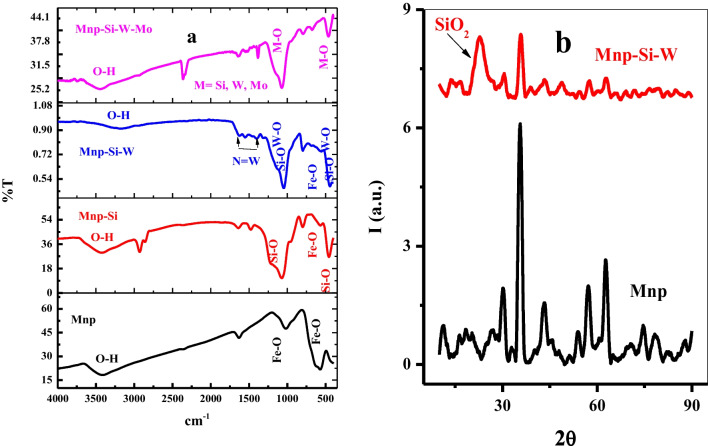
**a** FTIR spectrum of Mnp, Mnp-Si, Mnp-Si-W nanoparticles, and Mnp-Si-W-Mo complex. **b** XRD patterns of Mnp and Mnp-Si-W nanoparticles

To further follow the preparation procedure of Mnp-Si-W nanoparticles, FTIR spectra were performed. Figure 3 a presents the FTIR spectra of Mnp, Mnp-Si, and Mnp-Si-W nanoparticles. Mnp shows the characteristic peaks at 1020 cm^−1^ and 570 cm^−1^ related to Fe–O bond stretching. After coating the Mnp with silica, new bands were observed at 1074 cm^−1^, 799 cm^−1^, and 457 cm^−1^ for the SiO_2_-cored Mnp, which cross-correlated to symmetrical and asymmetrical vibrations of the Si–O–Si bonds and Si–O bond stretching, as well as bands at 2929 cm^−1^, which assigned the C–H stretching of surfactant residue used in the preparation process. In addition, the intensity of the band at 570 cm^−1^ referred to the Fe–O bond being highly reduced. In order to prepare Mnp-Si-W, Mnp-Si was modified with amino silane and then reacted with Na_2_WO_4_; therefore, the band at 3424 cm^−1^ (O–H) had shifted to 3168 cm^−1^. New bands in the range 1552–1314 cm^−1^ appeared, which may be related to N = W bond stretching. The band at 1074 cm^−1^ was shifted to 1048 cm^−1^ and the band at 457 cm1 was strengthened by Si–O and W–O stretching. After employing Mnp-Si-W in the adsorption of Mo ion, the intensity of the band due to O–H bond stretching shifted to 3447 cm^−1^ and became stronger. Moreover, the intensity of the band at 457 cm^−1^ becomes weak.

#### XRD

The XRD patterns of Mnp and Mnp-Si-W nanoparticles are shown in Fig. 3b. The Mnp exhibits diffraction peaks at 30°, 35.6°, 43.6°, 54°, 57°, and 62.8°, which excellently agree with the spinel structure corresponding to magnetite (Zhong et al. 2018). After shelling the Mnp with silica, a broad band at (2*θ* = 22.9°) was observed, which is characteristic of SiO_2_, and the bands at 30°, 35.6°, 43.6°, 54°, 57°, and 62.8° are highly weakening, which may be due to the amorphous silane shell composed around the Fe_3_O_4_ core.

#### BET surface area

**Fig. 4:**
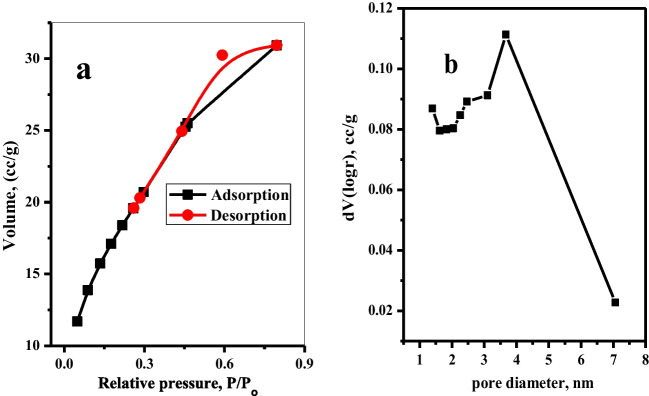
**a** Nitrogen adsorption–desorption isotherm at 77 K and **b** BJH pore size distribution curves of the Mnp-Si-W nanoparticles

The BET (Brunauer–Emmett–Teller) and BJH (Barrett–Joyner–Halenda) techniques, shown in Fig. 4 a and b, were used to determine the surface area and pore size distribution. The BET surface area, pore volume, and pore radius of Mnp-Si-W nanoparticles were determined and are tabulated in Table 3. The specific surface area of the Mnp-Si-W nanoparticles was found to be 66.54 m^2^ g^−1^. The BJH pore size distribution shows a pore size distribution with a center at 1.6 nm, confirming the microporous character of the current pores and including some mesopores in the range of 2–5 nm. The larger surface area of Mnp-Si-W nanoparticles affords more active sites, which will be useful for the Mo(VI) removal.

**Table 3:** BET surface area, pore volume, and pore radius of Mnp-Si-W nanoparticles

Sample	BET surface area (m^2^ g^−1^)	Average pore radius (nm)	Pore volume from BJH (cm^3^ g^−1^)	Pore radius from BJH (nm)	Total pore volume (cm^3^ g^−1^) for pore radius < 5.22 nm
Mnp-Si-W nanoparticles	66.54	0.144	0.052	0.69	0.048

#### EDX analysis

**Fig. 5:**
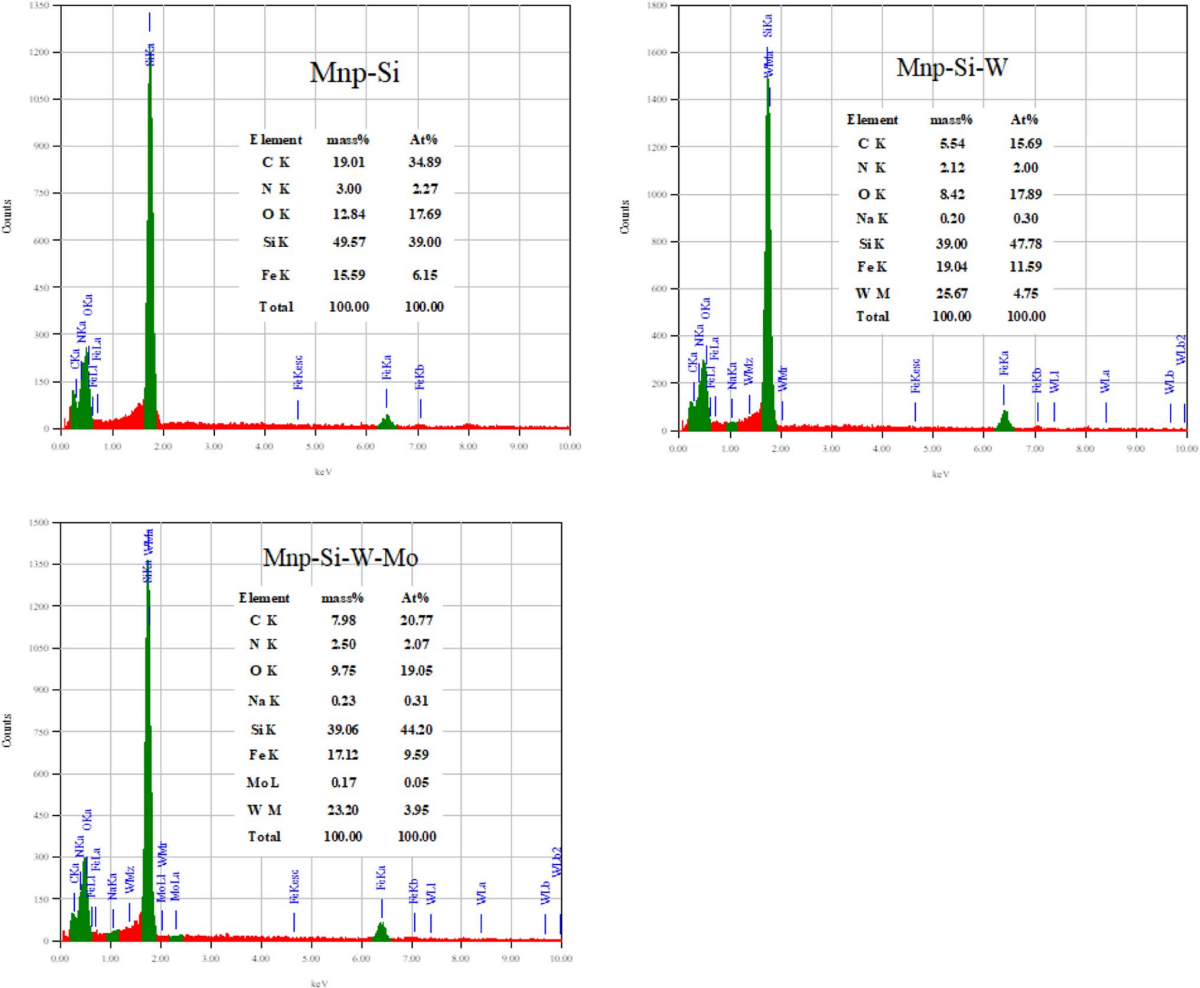
EDX analysis for Mnp-Si, Mnp-Si-W nanoparticles, and Mnp-Si-W-Mo complex

EDX analysis is valuable to identify the elemental composition of the prepared nanoparticle. The EDX analysis of the nanoparticle was evaluated before and after the sorption process. The EDX analyses of Mnp-Si, Mnp-Si-W, and Mnp-Si-W-Mo were studied as illustrated in Fig. 5. The EDX analysis of Mnp-Si and Mnp-Si-W showed C (amino silane), O (Map, SiO_2_, and Na_2_WO_4_), N (amino silane), Fe (Mnp), Si (TEOS, amino silane), and W (Na_2_WO_4_), as seen in Fig. 5. EDX analysis was also performed after the sorption process to investigate the successful sorption of Mo(VI) on the Mnp-Si-W nanoparticles.

### Adsorption investigation

#### Effect of adsorption solution pH

**Fig. 6:**
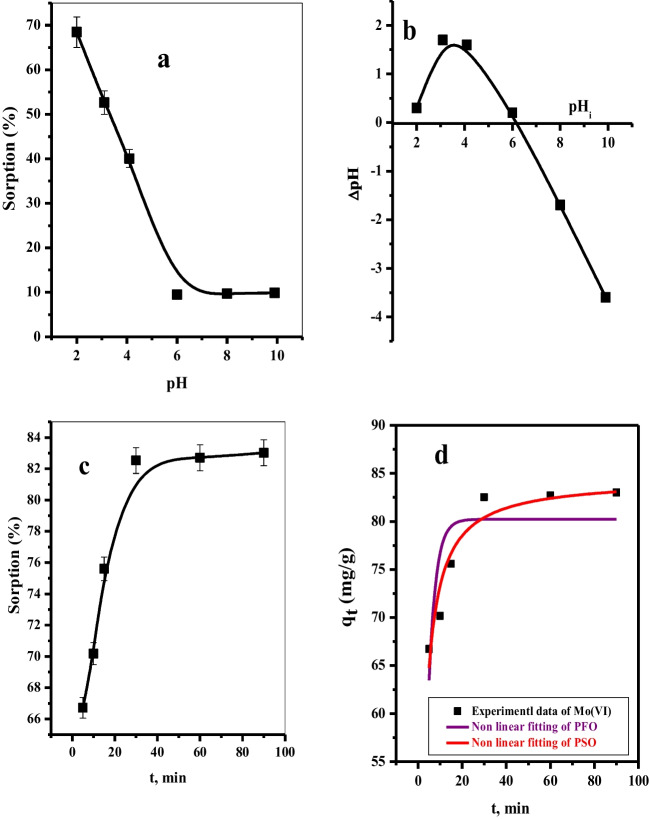
**a** Effect of the initial solution pH on the removal percentage (% R) of Mo(VI) from aqueous solution. **b** pH_ZPC_ of the Mnp-Si-W nanoparticles (*t* = 5 min, [Mo(VI)] = 100.0 ppm, dose = 5.0 mg, *V* = 5.0 mL, *T* = 25 °C). **c** Effect of contact time on sorption percent (% R) of Mo(VI) from aqueous solution. **d** Non-linear PFO and PSO models of Mo(VI) sorption ([Mo(VI)] = 100 mg L^−1^, dose = 5.0 mg, *V* = 5 mL, pH = 2, *T* = 25 °C) from aqueous media

The pH value of the solution is an important factor in the adsorption process, where it affected the surface properties of both adsorbent and adsorbate. In this section, we study the effect of pH values in the range of 2.0–10.0 on the adsorption percent of Mo(VI). Figure 6 a shows that the adsorbent achieves maximum Mo(VI) sorption (68.47%) at low pH (2), which linearly decreases to 9.48% at pH (6) and is still constant at pH (10). This behavior could be due to further changes in the pH of the aqueous solution; the Mo species will have differed as well as the charge on the adsorbent surface. However, in the pH range 2–4.6, Mo(VI) is present in various anionic polynuclear hydrolyzed species: Mo_7_O_21_(OH)_3_^−3^, Mo_7_O_22_(OH)_2_^−4^, Mo_7_O_23_(OH)^−5^, and Mo_7_O_24_^−6^ (Xiong et al. 2011a, b). Moreover, the zero of the point charge of the adsorbent is 6.2 (Fig. 6b). Consequentially, the surface-active sites of the adsorbent will be positively charged at pH < 6.2, which will be considered a suitable site for the adsorption of anionic species, and so, the adsorption percent will be enhanced. On the other hand, at pH > 6.2, the adsorption efficiency is highly diminished. This is attributed to the fact that the adsorbent binding sites will be negatively charged and compete with OH anions.

#### Effect of shaking time

The sorption of Mo(VI) on the adsorbent was tested as a function of shaking time in the range (5.0–90.0 min), as shown in Fig. 6c. We can note that the removal percent of Mo rapidly increased from 66.72 to 83.02% with an increase in the shaking time from 5.0 to 30.0 min. After which, the removal percent remained constant for 90.0 min. Two main non-linear kinetic models, the pseudo-first-order model and the pseudo-second-order model, were utilized to ascribe the adsorption process of Mo over Mnp-Si-W (see Fig. 6d). Various factors of these kinetic models and the correlation coefficient (*R*^2^) are summarized in Table 4 (Baral et al. 2008a, b). It can be noted that the PSO is (*R*^2^ = 0.999) close to the unit. Moreover, the calculated *q*_eCAL_ = 84.50 mg g^−1^ values also agree well with the experimental result *q*_e EXP_ = 83.02 mg g^−1^. These findings revealed that the PSO describes the Mo(VI) adsorption on Mnp-Si-W.

**Table 4:** Non-linear kinetic model parameter for adsorption of Mo(VI) from aqueous media ([Mo(VI)] = 100 mg L^−1^, dose = 5.0 mg, *V* = 5 mL, pH = 2, *T* = 25 °C)

Kinetic	Parameters	Metal ion
		Mo(VI)
Pseudo-first-order	*q*_e_ (mg g^−1^)	80.23
	*k*_1_ (g mg.min^−1^)	0.313
	*R*^2^	0.566
Pseudo-second-order	*q*_e_ (mg g^−1^)	84.50
	*k*_2_ (g mg.min^−1^)	0.656
	*R*^2^	0.903
*q*_exp_, mg/ g		83.02

#### Effect of initial metal ion concentration

**Fig. 7:**
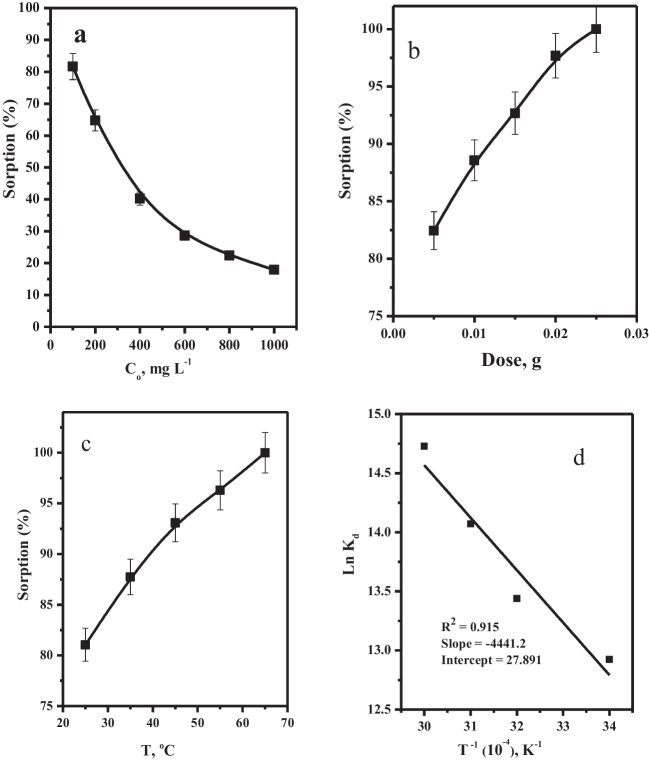
**a** Effect of initial metal ion concentration on the sorption percent of Mo(VI) from aqueous media (*t* = 30.0 min, dose = 5.0 mg, *V* = 5 mL, pH = 2.0, *T* = 25 °C using Mnp-Si-W nanoparticles. **b** Effects of adsorbent dose on the sorption percent of Mo(VI) from aqueous media. (*t* = 30.0 min, [Mo(VI)] = 100 mg L^−1^, *V* = 5 mL, pH = 2.0, *T* = 25 °C). **c** Effect of temperature on the sorption percent and **d** thermodynamic parameters of Mo(VI) from aqueous solution (*t* = 30.0 min, [Mo(VI)] = 100 mg L^−1^, dose = 5.0 mg, *V* = 5 mL, pH = 2.0, *T* = 25 °C)

Figure 7 a shows the impact of the initial Mo(VI) concentration on its adsorption percent in aqueous solutions. We noticed that as the initial Mo(VI) concentration was increased from 100.0 to 1000.0 mg L^−1^, the sorption efficiency of Mo(VI) decreased from 82.53 to 17.89%. This observation can be demonstrated as, at low Mo(VI) concentrations, the number of Mo(VI) species will be low compared with the number of binding sites on the surface of the adsorbent. This will result in enhancing the sorption percentage. However, as the Mo(VI) concentrations increase, the number of Mo(VI) species in the aqueous solution increases in front of a constant number of binding sites. This will lead to a direct reduction in removal efficiency.

### Effect of adsorbent dosage

According to Fig. 7b, the effect of adsorbent Mnp-Si-W nanoparticles on the sorption efficiency of Mo(VI) was investigated in the range of 0.005–0.025 g. The experimental results indicated that by increasing the dose of the Mnp-Si-W nanoparticles, the sorption efficiency of Mo(VI) was increased from about 82.44 to 99.9% as the adsorbent dosage increased from 0.005 to 0.025 g. This increase can be explained by the assumption that as the adsorbent dosage is increased, more active sites become available for the sorption of molybdenum species.

#### Effect of solution temperature

The effect of different temperatures on the sorption of Mo(VI) onto Mnp-Si-W nanoparticles was investigated in the range of 25.0–65.0 °C. As shown in Fig. 7c, when the temperature increased from 25.0 to 65.0 °C, the sorption percent increased from 81.05% to 99.9%. This is attributed to the fact that the increase in the solution temperature will enhance the movement of the Mo(VI) ions. Consequently, the collusion among the Mo(VI) ions and the binding sites will increase. As a result, the adsorption percent of Mo(VI) ions onto Mnp-Si-W will increase. This behavior exposed the endothermic nature of molybdenum’s sorption onto Mnp-Si-W nanoparticles. The thermodynamic parameters that include the standard free energy change Δ*G*°, the standard enthalpy change Δ*H*°, and the standard entropy change Δ*S*° for the sorption process can be calculated using the following equations (Elgoud et al. 2022)

6 $$\Delta G^\circ =-RT {\mathrm{ln }K}_{{\text{d}}},$$

7 $$\Delta G^\circ =\Delta H^\circ -T\Delta S^\circ$$

8 $${-RT\mathrm{ ln }K}_{{\text{d}}}=\Delta H^\circ -T\Delta S^\circ$$

9 $${\mathrm{Ln }K}_{{\text{d}}}=(\Delta S^\circ /R)+(-\Delta H^\circ /R)1/T$$

The standard thermodynamic parameters, including standard enthalpy change (Δ*H*°), standard free energy change (Δ*G*°), and standard entropy change (Δ*S*°), are illustrated in Table 5. The values of Δ*S*° and Δ*H*° can be determined from the intercept and slope of the plot of ln *K*_d_ versus 1/*T*, which would result in a straight line with a slope of (− Δ*H*°/*R*) and an intercept of (Δ*S*°/*R*) (Fig. 7d). The negative values of Δ*G*° indicate that the sorption reactions are feasible as well as spontaneous at all the studied temperatures. The positive value of Δ*H*° proves that adsorption is an endothermic type. In addition, the positive values of Δ*S*° indicate both a high level of randomization and a significant affinity of the Mnp-Si-W nanoparticles towards Mo(VI) ions during adsorption. The sorption reactions of Mo(VI) onto Mnp-Si-W nanoparticles can be deduced to be endothermic and spontaneous processes, and these results are consistent with previous studies with different adsorbents (Abu Elgoud et al. 2022b; Abd-Elhamid et al. 2024b; Abu Elgoud et al. 2023).

**Table 5:** Thermodynamic parameters for sorption of Mo(VI) ions

Metal ions	*T*, K	Δ*G*°, k J mol^−1^	Δ*H*°, k J mol^−1^	Δ*S*°, J mol^−1^ K ^−1^
Mo(VI)	298	− 32.18	36.92	231.89
	308	− 34.50		
	318	− 36.82		
	328	− 39.14		
	338	− 41.46		

#### Sorption isotherm and modeling

**Fig. 8:**
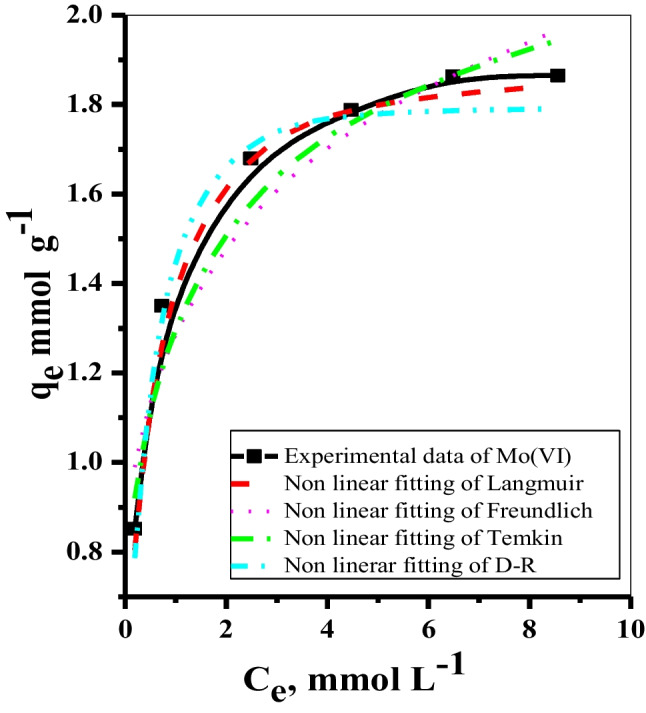
Non-linear fitting of the isotherm models investigated for the sorption of Mo(VI) from aqueous media, *t* = 30.0 min, dose = 5.0 mg, *V* = 5 mL, pH = 2.0, *T* = 25 °C using Mnp-Si-W nanoparticles

The adsorption equilibrium is explained by various adsorption isotherms, whose factors describe the adsorbent surface characteristics and their affinity towards the adsorbate. The experimental data were treated with various non-linear isotherm models. The values measured from the slopes and intercepts obtained from Fig. 8 for Mo(VI) using Mnp-Si-W are illustrated in Table 6. It was noticed that the Langmuir model owned the highest value of *R*^2^ = 0.986. This showed that the Langmuir isotherm model best described the adsorption equilibrium. The Mo species prefer to adsorb as a monolayer over equal energetic Mnp-Si-W active sites.

**Table 6:** Non-linear adsorption isotherm constants and *R*^2^ values for Mo(VI) sorption onto the Mnp-Si-W nanoparticles

Non-linear isotherm	Parameters	Metal ion
		Mo(VI)
Langmuir	*Q*_o_ (mg g^−1^)	182.03
	*Q*_max_ (mmol g^−1^)	1.9
	*K*_L_	3.842
	*R*^2^	0.986
Freundlich	*K*_f_	1.33
	*n*	5.541
	*R*^2^	0.921
Dubinin–Radushkevich	*q*_m_ (mg g^−1^)	172.26
	*q*_m_ (mmol g^−1^)	1.80
	*β*	0.246
	*R*^2^	0.934
	*E*_DR_	14.24
Tempkin	*A*_T_	159.07
	*B*_T_	0.27
	*R*^2^	0.921
*q*_exp_, mg/ g		**188.25**

#### Reusability and desorption of Mnp-Si-W nanoparticles

**Fig. 9:**
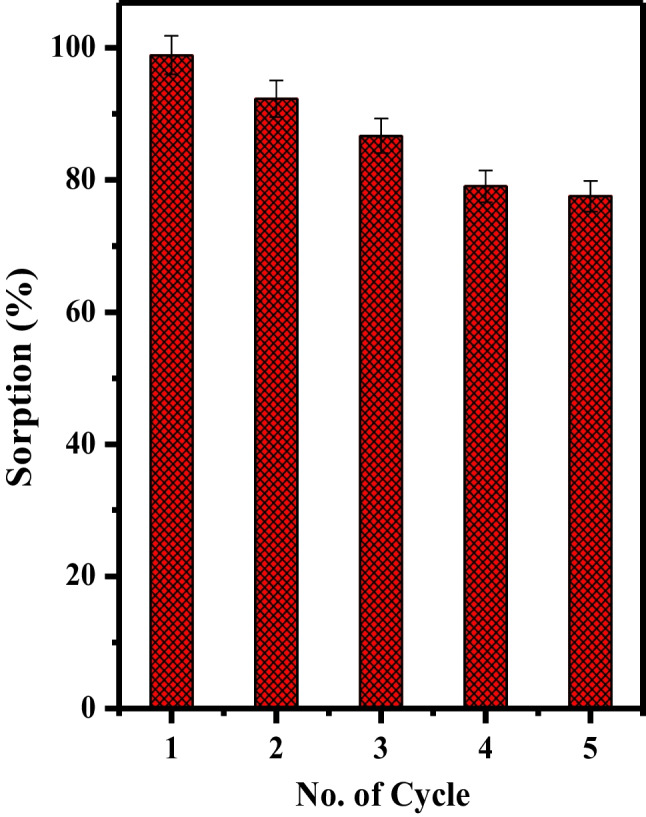
The effect of the number of the re-use cycles of the Mnp-Si-W nanoparticles on the sorption percentage of Mo(VI) (*t* = 30.0 min, [Mo] = 100 mg L^−1^, dose = 25.0 mg, *V* = 5.0 mL, pH = 2, *T* = 25 °C)

The regeneration capability of Mnp-Si-W nanoparticles was examined to make the adsorption process more economical and effective. Several reagents with a range of concentrations were used to recover Mo(VI) from loaded Mnp-Si-W nanoparticles. These reagents included sodium acetate, sodium hydroxide, and nitric acid. The desorption efficiency illustrated in Table 7 indicates that the best desorbing agent is 0.50 mol L^−1^ sodium acetate. The regeneration of the Mnp-Si-W nanoparticles was investigated by conducting five cycles of Mo(VI) adsorption and desorption experiments (see Fig. 9). The obtained results showed that the regeneration of the Mnp-Si-W nanoparticles was highly effective.

**Table 7:** Desorption of Mo(VI) from loaded Mnp-Si-W nanoparticles

Stripping agent	Desorption percent (%)
	Mo(V)
HNO_3_ (mol L^−1^)	
0.5	0
1.0	0
Sodium hydroxide (mol L^−1^)	
0.5	55.35
1.0	40.58
Sodium acetate (mol L^−1^)	
0.50	98.80
1.0	99.9

*V*/*m* = 1.0 L g^−1^, shaken time = 60.0 min, *T* = 25 ± 1 °C

#### Selectivity of Mnp-Si-W nanoparticles

In order to determine the selectivity of Mnp-Si-W nanoparticles towards Mo(VI) ions, the sorption of Mo(VI) ions was investigated in the presence of a mixture of other metal ions such as Y(III), Sr(II), Cs(I), Eu(III), and La(III). For this purpose, Mnp-Si-W nanoparticles were added to an aqueous solution with initial concentrations (100 mg L^−1^) of each ion (Y(III), Sr(II), Cs(I), Eu(III), and La(III) and Mo(VI) ions) of pH 2.0 and shaken for 30.0 min and *V*/*m* = 0.10 L g^−1^. After that, the final concentration of each M-ion was determined. The values for the distribution coefficient and separation factor are tabulated in Table 8. As shown in Table 8, Mnp-Si-W nanoparticles had much higher selectivity for molybdenum in the presence of some simulated fission product elements.

**Table 8:** Selectivity of Mnp-Si-W nanoparticles toward Mo(VI)

Ions	Sorption %	*K*_d_ (L g^−1^)	Separation factor *SF*_Mo/M_
Mo(VI)	61.25	0.158	–
Eu(III)	27.06	0.037	4.27
La(III)	22.37	0.029	5.45
Y(III)	21.97	0.028	5.64
Sr(II)	13.16	0.015	10.53
Cs(I)	4.26	0.0044	35.91

#### Effect of interfering anions on the sorption of Mo(VI)

The effect of different individual anions such as sulfate, phosphate, and chromates on the sorption of Mo(VI) ions was studied in the range of different concentrations from 0.0 to 0.25 mol L^−1^ under the optimum conditions (a shaking time of 30.0 min, a pH of 2.0, and a *V*/*m* of 1.0 L g^−1^), and the obtained data are listed in Table 9. It is noted that the sorption percent of Mo(VI) decreased with an increase in the concentration of interfering anions in the order phosphate < sulfate < chromate. In the presence of phosphate, the removal of Mo(VI) decreased from 79.80 to 50.31%, while the existence of sulfate will result in decreasing the sorption percent of Mo(IV) from 79.80 to 48.32%. Moreover, interfering with chromate ions will highly decrease the sorption percent of Mo(VI) from 79.80 to 25.58%.

**Table 9:** Effect of interfering ions on the sorption of Mo(VI)

Interfering ions	Concentrations, mol L^−1^	Adsorption percentage (%)
Phosphate	0.0	79.80
	0.01	73.92
	0.05	62.21
	0.1	55.23
	0.25	50.31
Sulfate	0.0	79.80
	0.01	65.52
	0.05	60.46
	0.1	54.46
	0.25	48.32
Chromate	0.0	79.80
	0.01	42.34
	0.05	33.85
	0.1	28.78
	0.25	25.58

#### Adsorption mechanism

**Fig. 10:**
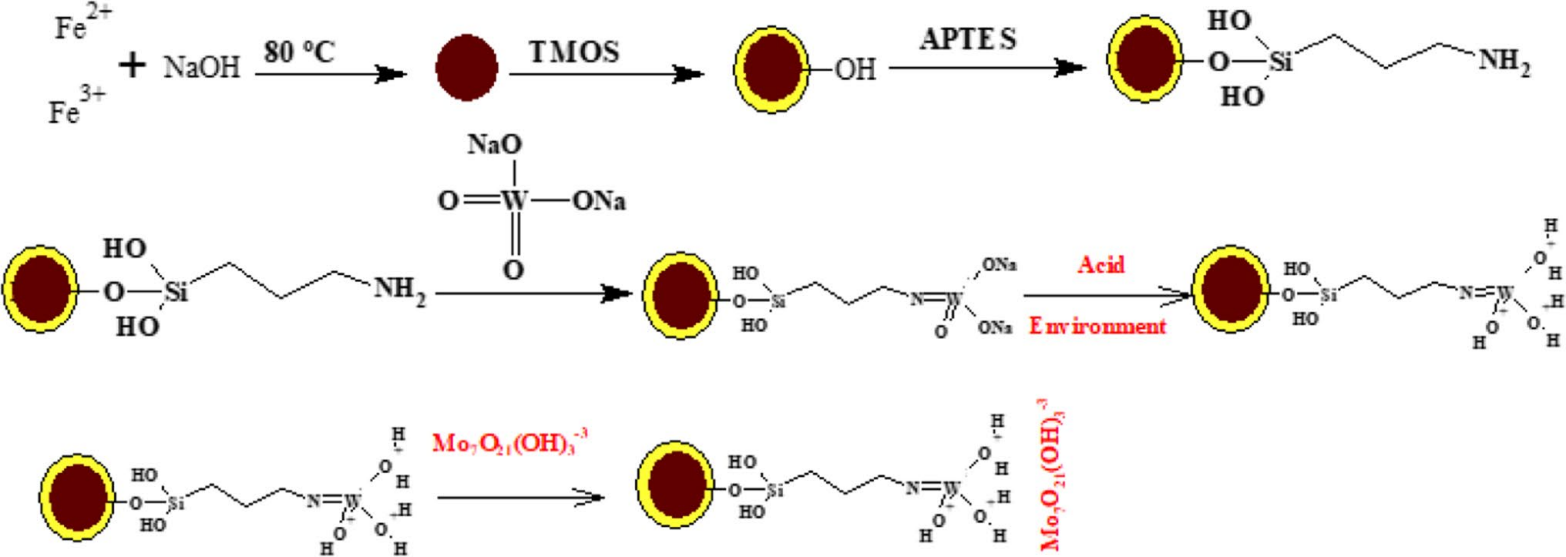
Adsorption mechanism of Mo-anion

As shown in the following figure (Fig. 10), the tungstate was chosen due to it has the ability to link with the amino silane-modified magnetic nanoparticles through the W = O bond. In an acidic environment, the other oxygen atoms will be protonated and the prepared (Mnp-Si-W) will be positively charged as indicated from the point of zero charge (the “Effect of adsorption solution pH” section; Fig. 6a, b). Moreover, with an increase in the number of O-atoms, the number of positive sites will be increased. Consequently, the affinity of the material towards the anion species will be increased, and the adsorption efficiency will increase. On the other hand, in an acidic medium, Mo(VI) is present in various anionic polynuclear hydrolyzed species, Mo_7_O_21_(OH)_3_^−3^, Mo_7_O_22_(OH)_2_^−4^, Mo_7_O_23_(OH)^−5^, and Mo_7_O_24_^−6^, which were suitable for interaction with positively charged active sites located at the Mnp-Si-W nanoparticle surface. This is confirmed by the enhancement of the adsorption percent of Mo(VI) ions by decreasing the value of the pH (refer to the “Effect of adsorption solution pH” section; Fig. 6a, b).

#### Comparison of the investigated Mo(VI) ions onto various adsorbents

The difference in sorption capacity between various adsorbent materials is due to the characteristics of each adsorbent, such as porosity, functional groups, and particle size. Table 10 compares the sorption capacity of Mnp-Si-W nanoparticles with various adsorbents from the literature. The results showed that, in comparison to other magnetic adsorbent materials, Mnp-Si-W nanoparticles have a better capacity for molybdenum sorption. In order to adsorb and separate Mo(VI) from aqueous solutions, Mnp-Si-W nanoparticles can be used as a highly effective adsorbent.

**Table 10:** Comparison of capacity values for Mo(VI) adsorbed by several adsorbents

Metal ion	Adsorbent	*Q*_o_, mg g^−1^	Ref
Mo(VI)	Mnp-Si-W nanoparticles	**182.03**	**This work**
	Magnetic Cr-ferrite	26.8	Gamal et al. (2021)
	Montmorillonite resin	162.0	Tuchowska et al. (2019)
	D201 resin	85.0	Namasivayam and Sureshkumar (2009)
	D290 resin	76.3	Liansheng et al. (2001)
	TVEX-TOPO resin	17.5	Masry and Daoud (2021)
	Ion-imprinted polymer (Mo(VI)-MIIP)	28.0	Hassanpour and Taghizadeh (2016)
	Nano-magnetic CuFe_2_O_4_	30.58	Tu et al. (2014)
	NaOCl-oxidized multiwalled carbon nanotubes	22.73	Chen and Lu (2014)
	Maghemite nanoparticles	33.4	Afkhami and Norooz-Asl (2009)
	Ca-alginate/Lix-84 beads	72.2	El-Din et al. (2021)
	Fe_3_O_4_/chitosan nanoparticles	35.5	Chao et al. (2021)
	D301 resin	157	Guo et al. (2021)
	Modified D301 resin	428	Guo et al. (2021)
	Impregnated perlite	18.51	Rizk et al. (2018)

## Conclusion

The tungstate-modified magnetic nanoparticle was successfully synthesized and applied to remove molybdenum from aqueous solutions. The as-prepared nanoparticle was characterized using TEM, FTIR, BET, XRD, and EDX. The batch mode was utilized to study the activity of the prepared materials towards Mo(VI). The experiment results showed that the adsorption process follows the pseudo-second-order. Moreover, according to the maximum adsorption isotherm, the maximum removal capacity was found to be 182.03 mg g^−1^ for Mo(VI) at pH 2.0. Thermodynamic values indicated that the sorption process was an endothermic and spontaneous reaction. Moreover, the magnetic nanoparticle modified with tungstate is a suitable candidate for the selective sorption of Mo(VI) from some fission products. On the other hand, the presence of anions highly retards the removal of Mo(VI) on order chromate > sulfate > phosphate. Finally, the removal percentage was reduced by ≈ 12% after five times of adsorption–desorption. Further work will be addressed to investigate the sorption behavior of Mo(VI) using the newly developed materials using the fixed column technique.

## Data Availability

All data generated or analyzed during this study are included in this published article.
